# The Role of Medication in Supporting Emotional Wellbeing in Young People with Long-Term Needs

**DOI:** 10.3390/healthcare5040084

**Published:** 2017-11-03

**Authors:** Nicola J. Gray, Damian M. Wood

**Affiliations:** 1 Green Line Consulting Limited, Manchester M28 7FF, UK; 2 Nottingham Children’s Hospital, Nottingham University Hospitals NHS Trust, Queen’s Medical Centre, Nottingham NG7 2UH, UK; damian.wood@nuh.nhs.uk

**Keywords:** adolescent, young adult, young person, medication, adherence, mental health, physical health, long-term condition, emotional wellbeing, health services

## Abstract

Young people frequently use and access prescribed medications for a range of health problems. Medications aimed at treating both common health problems and long-term physical and mental health needs in adolescence can have a significant effect on a young person’s emotional well-being. We use a series of case studies to illustrate the challenges for healthcare professionals supporting young people with medication use. The studies illustrate the efficacy and limitations of medication on improving emotional well-being by alleviating illness and distress, and how this efficacy must be balanced against both the adverse effects and the burden of treatment. There are specific challenges for medication management during adolescence including issues of adherence/concordance, facilitating autonomy and participation in decision making, and promoting independence.

## 1. Introduction

The Health Survey for England 2013 reported that 14% of young men and 25% of young women aged 16–24 years had taken prescription medicines in the previous week [1]. The most commonly used medicines by this age group included those for both physical and mental health problems—medicines for respiratory conditions, antidepressants, antibacterials, analgesics, and/or non-steroidal anti-inflammatory drugs (NSAIDs). It is likely that the frequency of medicine use that includes non-prescription medicines for short-term health problems would make this figure significantly higher. We can conclude that a significant proportion of young people are familiar with the use of medicines. Some medicines address the rapid treatment of symptoms, such as reliever inhalers for asthma or non-prescription painkillers for headache; other medicines must be taken whether symptoms are present or not to achieve long-term maintenance of conditions and to minimise breakthrough symptoms, such as anti-rejection transplant medicines or pancreatic enzyme supplements for cystic fibrosis. Moreover, many young women take daily oral contraceptives or use a long-term implant to prevent unplanned pregnancy.

We assert in this paper that emotional wellbeing among young people will be supported by the effective use of medication to alleviate symptoms of both physical and mental health conditions that might otherwise be seen as lifestyle-limiting. Equally, we recognise that the burden of treatment of a long-term condition and the need to adhere to medication routines during adolescence may result in the rejection of a long-term treatment, with potential consequences for the health and well-being of the young person. The finding that young people have lower rates of adherence to medication than either children or adults, and associated risk factors, has been reported in the literature although evidence is sparse and inconsistent [2,3,4]. A review of qualitative literature about the lived experience of children and young people with asthma, diabetes, and epilepsies explored the theme that treatment regimens may be constraining or enabling [5]. Work in the context of juvenile arthritis shows similar themes [6,7]. All too often the medicine regimen dominates the life of the young person, rather than being created to fit within the existing life routine and interests of the individual [8]. Suris et al. reflected on the often-contradictory findings of reviews and studies of the impact of a long-term condition on mental health and wellbeing, but they asserted that “…the wellbeing of chronically ill adolescents is determined largely by the severity of the disease and the amount of treatment required” [9].

The aim of this paper is to explore the role of medicines in supporting emotional wellbeing among young people with long-term health needs, which includes diagnosed physical and mental health conditions. Our scope will include the use of all medicines to their best effect to control conditions that might otherwise cause distress, and the use of psychotropic medicines whose primary mode of action is to enhance emotional wellbeing or to control symptoms that might undermine wellbeing. 

## 2. Materials and Methods

In this paper, we will present three case studies that illustrate a range of issues that are relevant to the use of medicines by young people to support emotional wellbeing. These cases derive from both physical and mental health long-term needs. The cases reflect our combined clinical and research experience. The third case study, about mental health issues, is adapted from a series of real-life experiences of young people taking psychoactive medicines from the UK ‘HeadMeds’ website, hosted by the charity YoungMinds (Appendix A).

## 3. Results

### 3.1. Case Study 1—Acne

Imogen is 15 years of age and has moderate acne affecting her face, neck, back, and chest. She has tried a range of prescribed and over the counter treatments over the last two years, including combined topical acne treatments in conjunction with oral antibiotics. A recent trial of a combined oral contraceptive pill has failed to improve her acne. She has some early scarring on her cheeks. Her acne is having a significant impact on her emotional well-being and has started to impact on her self-esteem, confidence, social activities and mood. She presents to the dermatology clinic with her mother seeking oral isotretinoin treatment.

Oral isotretinoin is an effective treatment for moderate–severe acne that has failed to respond to combined topical acne and oral antibiotic therapy and is an example of a pharmacological treatment of a physical condition which is largely aimed at improving psychological wellbeing [10,11]. This impact applies for the short-term effects of having acne and the longer-term effects of scarring. However, isotretinoin has been associated with low mood, and there have been concerns regarding suicidality and deaths [12]. For many young people, acne can have a significant detrimental effect on their emotional wellbeing and mental health [13]. When considering oral isotretinoin treatment the dermatologist, the young person and family need to balance the risks of treatment with the risks of failing to treat the acne adequately. The impact of acne on the young person is often underestimated and careful assessment of mood, expectations, and concerns about treatment need to be explored prior to initiating treatment. Isotretinoin is highly teratogenic and pregnancy prevention is a priority during isotretinoin treatment [14]. Discussions regarding sexual activity, pregnancy prevention, and contraception need to run alongside discussion of mood and other aspects of treatment; this is especially important, given the impact of an unplanned pregnancy with a high risk of foetal malformation on the well-being of both the young person and the unborn child.

### 3.2. Case Study 2—Chronic Headache

Kyle is 14 years old and has chronic daily headaches. The headaches have been present for the past three months and are affecting his school attendance, as well as family and social activities. Examination and investigations including brain imaging has not revealed a cause and the headaches are getting worse. A headache diary reveals a constant daily headache occurring on most days, with no features to suggest migraine or cluster headaches. Kyle has been using daily doses of over-the-counter paracetamol and ibuprofen products. In addition, he has been taking some of his mother’s prescribed codeine-acetaminophen(paracetamol) tablets for severe headaches. Kyle’s headaches are affecting his emotional wellbeing; he is irritable, and starting to isolate himself from his friends. He describes his mood as low and cannot enjoy activities because of the pain.

Chronic headache is a common problem in young people and can have a significant impact on their emotional wellbeing [15]. Medication overuse headache is an example where pharmacological treatment of a problem may have unintended consequences if use is prolonged [16]. In this case, worsening headache occurred through the use of over-the-counter analgesics. Guidance for the management of headache in young people reminds us to be alert to the possibility of medication overuse headache in people whose headache developed or worsened, while they were taking paracetamol, or an NSAID, either alone or in any combination, on 15 days per month or more for 3 months or more. The UK National Institute for Health and Care Excellence (NICE) recommends abrupt rather than gradual withdrawal of the overused medication for one month, and consideration of a preventative treatment [17]. There are few effective pharmacological therapies for those with headaches, which are not migrainous in nature. The natural history of chronic daily headache is remission with time; however, early onset, longer duration, anxiety, and medication overuse predict persistence at follow-up [18,19,20]. The relationship between chronic headache and psychological comorbidity is well reported but poorly understood [21]. For chronic tension type headaches, the UK NICE recommended preventative treatment is to consider a course of up to 10 sessions of acupuncture over 5–8 weeks for the prevention of chronic tension-type headache. The message here therefore is that, for the treatment of chronic (tension) headache in young people, ‘less is sometimes more’ when it comes to medication.

### 3.3. Case Study 3—Depression

Zoe is 19 years old. She was diagnosed with depression at the age of 16 and was prescribed fluoxetine, a selective serotonin reuptake inhibitor (SSRI) antidepressant. She says that she had felt sad and had a low mood. She found that the fluoxetine did lift her mood, but it also caused her to gain weight so she stopped the medication without consulting the prescriber and her weight went back to normal. She feels that she had no discontinuation symptoms, but she says that her mother noticed she was grumpier when she stopped this medicine. More recently, Zoe has started to take sertraline, another antidepressant. She feels that taking medication has improved her relationships, noting that she is less grumpy with her family when taking it. She has had some side-effects from the sertraline—it made her feel ‘shaky’—but she reflects that this does not affect her life dramatically enough for it to become a barrier to taking it. The positive effects outweigh this.

This case study shows the use of psychoactive medication to treat symptoms that are directly affecting emotional wellbeing. Zoe’s first medicine was fluoxetine, which at the time of writing this paper was the only antidepressant with UK marketing authorisation for use in this indication for children and young people aged 8–18 [22]. UK NICE guidance published in March 2015 indicates fluoxetine for first-line use in moderate to severe depression for 12–18 year-olds [23]. The use of antidepressant medication in young people aged under 18 years is, however, discouraged. The British National Formulary for Children simply asserts that “[t]he balance of risks and benefits for the treatment of depressive illness in individuals under 18 years is considered unfavourable”, and further reflects that “[c]linical trials have failed to show efficacy and have shown an increase in harmful outcomes” [24]. A systematic review of published and unpublished RCT data about SSRIs, published in 2004, concluded that “risks could outweigh benefits of these drugs (except fluoxetine) to treat depression in children and young people” [25].

Weight gain is not listed as a side-effect of fluoxetine; conversely, reports of reduced appetite and subsequent weight loss are noted by the manufacturer and supported by the literature [26]. The importance of the risk of weight gain is underlined, however, by the fact that Zoe was willing to abandon a medicine that was improving her mood because of the impact on her body image. Tremor is a common side-effect of antidepressant medications [22]. The acceptance of tremor as a reasonable trade-off for the benefit of the medication on mood indicates the careful consideration of harm–benefit issues by the young person and the prioritisation of adverse effects relating to weight.

## 4. Discussion

In order to achieve the best effect from medication for young people, thus supporting emotional wellbeing, it is important to consider different aspects of medication use that potentially enhance or undermine its benefits. Our case studies in this paper have been chosen to stimulate reflection about the use of medication in different scenarios. The goal of all medication use, whether for a physical or mental health condition, should be to maximise physical and emotional wellbeing in parallel; however, the burden of illness and the efficacy of the medication must be balanced against the potential adverse effects and the burden of treatment. If a physical health condition such as acne is treated effectively with relevant medication, this can have a significant positive effect on emotional wellbeing; highly effective treatments such as isotretinoin, however, have the potential for serious adverse effects on emotional wellbeing. Conversely, clinicians must remain alert to the overuse of non-prescription or prescribed medication that may contribute to ill health and impair emotional wellbeing.

The case studies highlight some cross-cutting issues that should be borne in mind by any clinician practicing in this field. Careful attention must be paid to the priority that young people assign to both the positive and negative effects of their medication. No assumptions must be made about the value that an individual might assign to these effects. Even if a medicine is achieving a good therapeutic effect on the primary symptoms, such as reducing the incidence of acne or lifting mood, there may be other negative effects that override the benefit, such as weight gain or future effects on fertility. Encouraging individuals to explicitly state their highest priorities could help to negotiate an acceptable regimen.

Nonadherence to any medicine should be normalised for young people. Very few people, including health professionals, can take every dose of every medicine they have without some slips. It is vital to distinguish between unintentional and intentional nonadherence, as the strategies for moving forward will be very different [27]. Unintentional nonadherence may result from an inability to get the medicine or administer it, and very practical measures might solve the problem such as changing the type of asthma inhaler device. Intentional nonadherence may be particularly rooted in perceived negative impact on overall wellbeing that will need sensitive discussion. The clinician must accept that the regimen may not ultimately be sustainable and not impart blame for this.

Discontinuation of medication without prior consultation should be anticipated for all new regimens. Clinicians may wish to discuss this at the point of first prescription with the young person and family, framing it as part of the independence and autonomy of the young person that they have the right not to take it. For some medicines, sudden discontinuation may have more profound effects than others – this should be highlighted where there are potential problems of withdrawal effects.

Anticipating common side-effects, and giving useful advice about what to do if they happen, should be an integral part of consultations with young people and families, as this is known to be a concern for young people [28]. Similarly, if the full effect of a medicine is likely to take some time to emerge, as with antidepressants, young people should be prepared for this. Asking young people and families to persist with these therapies for an agreed time, accompanied by an effective explanation of why this happens and by proactive follow-up, may reduce the chances of early discontinuation. An honest conversation explaining that the young person may have to try several different therapies before they find the right one for their particular circumstances should be explained and accepted at the beginning of treatment to avoid feelings of frustration and failure by both parties. In this way, expectations can be managed, and the clinician and the family can unite in the search for the best option for that individual.

The use of medicines off-licence must be done thoughtfully, recognising the extra responsibility taken on by the clinician. Young people and families should be aware if a medicine has not been licensed in their age group. It is true that most medicines used in young people aged under 18 have been supported by clinical use in practice rather than randomised controlled trials. Promoting the voice of young people and families in the medicine licensing process and advocating for more clinical trials for this age group [29] are long-term strategies to reduce this knowledge gap [8].

Some research, education, and policy papers have explored shared decision-making between young people, parents/caregivers and prescribers across a range of physical and mental health conditions [30,31,32], indicating that passive decision-making is also an active choice [33,34]. In the context of mental health medicines, O’Brien et al. have suggested that “participation in treatment decisions can prepare youth for making their own decisions as adults, can be therapeutic, and can have positive effects on their self-confidence and self-esteem” [35]. As the young person develops their expertise with their condition, maintenance requires a level of commitment that may also be supported by family members, friends, and the members of their increasingly multidisciplinary healthcare team. Reliable and accessible information about medicines that is aimed specifically at young people, however, is not plentiful—the Headmeds website was created to try and address this gap for psychoactive medicines. 

The potential of family members and friends as observers and supporters for young people should be appreciated. All the case studies refer to the input of a family member. Whilst independent consultation with the young person affords an opportunity to explore their feelings about their condition and medicines without their family members present, the clinician should also, with the permission of the young person, enhance parents’/carers’ knowledge about the medication so they can act as an extra resource for the young person. As the young person approaches adulthood, they will inevitably assume more responsibility for their own health [36]; however, the provision of developmentally appropriate healthcare [37] can ensure that these transitions are managed and do not result in poorer outcomes.

Future research should explore the satisfaction of young people with the effectiveness of their medication for their physical or mental health condition, and its impact on their emotional wellbeing. Studies should also explore the assessment of relative harm and benefit by young people to help clinicians to have open and effective conversations with young people and families. The evidence base from clinical trials for all medicines intended for use by young people must continue to be improved, coupled with encouragement to young people to report adverse drug reactions, to maintain safety and wellbeing. 

## 5. Conclusions

Thoughtful use of medication can support emotional wellbeing among young people with long-term needs. More consideration needs to be given to the parity between services for physical health conditions, where medicines are considered central to management, and mental health conditions where the use of medication might be seen as a last resort or failure. Shared decision-making with young people and families, with honest discussion and relevant information, is essential to see benefit. In this way, medication can be tailored to the life context of the young person, consistent with their goals and priorities.

## Appendix A

In 2014 Young Minds, a UK mental health charity, launched the ‘Headmeds’ website where young people can access information about a number of commonly used mental health medicines. The website provides general information about these medicines to young people in the United Kingdom. The information is drawn from the summary of product characteristics (SPC) and the patient information leaflet (PIL) associated with each medicine from the Electronic Medicines Compendium (eMC) website, https://www.medicines.org.uk/emc/, which is a repository of statutory information for medicines licensed in the UK. The statutory information has been translated by specialist pharmacists to make it relevant to the lives of young people. There are 32 narratives on the Headmeds website section entitled ‘My Story’, which can be found at http://www.headmeds.org.uk/my-story. Young people were invited through the website, and through the networks of the Young Minds charity, to share their experience. Each contributed story may include a video, which may feature the young person telling the story or an actor working from the real-life story script. For Case Study 3, we have used the summary written text featured on one story page as the primary data source, with the permission of Young Minds.
